# 5-HT_1A_ receptor agonism in the basolateral amygdala increases mutual-reward choices in rats

**DOI:** 10.1038/s41598-020-73829-z

**Published:** 2020-10-06

**Authors:** Lisa-Maria Schönfeld, Sandra Schäble, Maurice-Philipp Zech, Tobias Kalenscher

**Affiliations:** Comparative Psychology, Institute of Experimental Psychology, Heinrich Heine University Düsseldorf, Universitätsstraße 1, 40225 Düsseldorf, Germany

**Keywords:** Amygdala, Motivation, Reward, Social behaviour, Social neuroscience, Neurotransmitters

## Abstract

Rats show mutual-reward preferences, i.e., they prefer options that result in a reward for both themselves and a conspecific partner to options that result in a reward for themselves, but not for the partner. In a previous study, we have shown that lesions of the basolateral amygdala (BLA) reduced choices for mutual rewards. Here, we aimed to explore the role of 5-HT_1A_ receptors within the BLA in mutual-reward choices. Rats received daily injections of either 50 or 25 ng of the 5-HT_1A_ receptor agonist 8-OH-DPAT or a vehicle solution into the BLA and mutual-reward choices were measured in a rodent prosocial choice task. Compared to vehicle injections, 8-OH-DPAT significantly increased mutual-reward choices when a conspecific was present. By contrast, mutual-reward choices were significantly reduced by 8-OH-DPAT injections in the presence of a toy rat. The effect of 8-OH-DPAT injections was statistically significant during the expression, but not during learning of mutual-reward behavior, although an influence of 8-OH-DPAT injections on learning could not be excluded. There were no differences between 8-OH-DPAT-treated and vehicle-treated rats in general reward learning, behavioral flexibility, locomotion or anxiety. In this study, we have shown that repeated injections of the 5-HT_1A_ receptor agonist 8-OH-DPAT have the potential to increase mutual-reward choices in a social setting without affecting other behavioral parameters.

## Introduction

Rats are social: they live in colonies with clear social relationships^1^, they cooperate^2^, they learn strategies by observing others^3^, they adapt their behavior in the presence of another rat^4^ and they even show behavior potentially motivated by empathy^5^. In a previous experiment by our group, we have shown that rats exhibit mutual-reward preferences, i.e., they prefer options that yield a reward to both themselves and a partner rat to options that only benefit themselves, but not the partner^6^. Since the choice for a mutual reward does not lead to any direct benefit for the rat that makes the decision, it is assumed that a preference for mutual rewards reflects rodent social behavior, specifically prosocial decision making^7,8^.

Several brain regions are known to contribute to the complex behaviors involved in sociality. One of these regions is the amygdala, which has been involved in social behavior in various species^9–12^. Lesions of the basolateral amygdala (BLA) induced in neonatal rats lead to disturbed social behavior during adulthood^13^ and BLA lesions induced in adult rats impair social behavior by eliminating mutual-reward preferences in rats^14^. These lesion studies have provided initial insight into the involvement of the amygdala, specifically the BLA, in social behavior. However, little is known about the psychopharmacological processes in the BLA that underlie social behavior. One candidate neurotransmitter that might play a relevant role in the modulation of social behavior within the BLA is serotonin. In general, serotonin is involved in affective and social behavior of various species, for example depression^15^, cognitive control of emotions^16^ and aggression^17^. In humans, lower levels of serotonin have been linked to a higher rejection rate of unfair offers and an increased likelihood to punish unfair behavior, whereas higher levels of serotonin led to an increased awareness of the well-being of others^18–20^. In rats, higher levels of serotonin decrease social play, whereas serotonergic lesions lead to divergent effects of social play behavior based on the dominance status of the respective rat^21,22^. In primates, the acquisition of a dominant status can be influenced by serotonergic drugs^23^. In addition, endogenous serotonin levels were found to be altered in the rat amygdala, prefrontal cortex, cingulate cortex and nucleus accumbens following maternal isolation or social isolation from conspecifics^24–26^.

The amygdala is densely innervated by serotonergic afferents that originate in the dorsal raphe nucleus and synapse in the lateral, basolateral and central nuclei of the amygdala (as reviewed by Asan et al.^27^). In the BLA, the majority of interneurons and pyramidal neurons express inhibitory 5-HT_1A_ receptors^28,29^. Intra-BLA injections of an agonist for the 5-HT_1A_ receptor induced anxiolytic and anti-panic effects in rats^30^, whereas a 5-HT_1A_ receptor antagonist prevented the impairing effects of stress on memory^31^.

The high concentration of 5-HT_1A_ receptors in the BLA together with the number of studies suggesting a specific role of 5-HT_1A_ receptors in cognitive and emotional functioning and social interaction^32–35^ leads to the question if 5-HT_1A_ receptors in the BLA are relevant for mutual-reward preferences and how these preferences are altered upon activation of 5-HT_1A_ receptors. Additionally, since the acquisition and expression of social preferences are shown to be distinct phenomena^6,7^, we aimed at dissociating the effects of 5-HT_1A_ receptor agonist injections into the BLA on the acquisition versus expression of mutual-reward choices.

## Materials and methods

### Subjects

This experiment was conducted according to the European Union Directive 2010/63/EU for animal experiments and further approved by German authorities (Landesamt für Natur, Umwelt und Verbraucherschutz). Four batches of twelve rats each (Charles River, Italy), about 10 weeks of age upon arrival, were housed in groups of three under a reversed 12 h day/night cycle. The housing room was kept at a constant temperature of 22 °C and a humidity of approximately 60%. Before and after surgery, rats received standard laboratory rodent food (Sniff, Germany) and water ad libitum. During the behavioral training and testing period, rats were food-restricted to 85% of their free-feeding body weight. Within each batch of rats, the cage containing the lightest three rats was designated as the partner-cage and the cages containing the remaining nine rats were designated as actor-cages (see below for a definition of actor and partner rats). We assigned the lightest rats as partner rats because we have shown that mutual-reward preferences are stronger when actors were paired with a lighter partner^6^.

### Surgical procedures

All actor rats (36 rats in total) underwent implantation of bilateral cannulas targeting the BLA. Prior to surgery, rats received analgesia (5 mg/kg carprofen s.c.), anesthesia was induced with 5% isoflurane until rats became immobile and then lowered to 1.5–3% isoflurane for maintaining anesthesia. Upon loss of the paw pinch reflex, the head was shaved and rats received multiple injections of a local anesthetic at the incision side and behind the ears (0.5 mg bupivacaine s.c.). Shortly after, rats were fixed in a stereotactic frame using blunt earbars (David Kopf Instruments, USA), had their eyes covered with ointment to prevent dehydration (Bepanthen, Bayer, Germany) and received an anal temperature probe connected to a heating pad to ensure a constant body temperature of 37 °C during the whole surgical procedure. The skull was exposed and bilateral holes were drilled at AP 2.5 mm posterior to bregma and ML 4.8 mm lateral to bregma. Guide cannulas (infusion guide cannula 313G, PlasticsOne, USA) were lowered 8.6 mm deep to target the BLA and fixed to the skull using dental cement (Paladur, Kulzer, Germany). To prevent obstruction, guide cannulas were sealed using removable dummy needles having the same length as the cannulas. The incision was sutured with one stitch at the front and back of the cannulas and rats were left to recover for 1 week, receiving analgesia (5 mg/kg carprofen s.c.) during the first 2 days after surgery. In total three rats died; two died under anesthesia during surgery and one was euthanized because it fulfilled standardized criteria for a humane endpoint^36^.

### Pharmacological treatment

**Figure 1:**
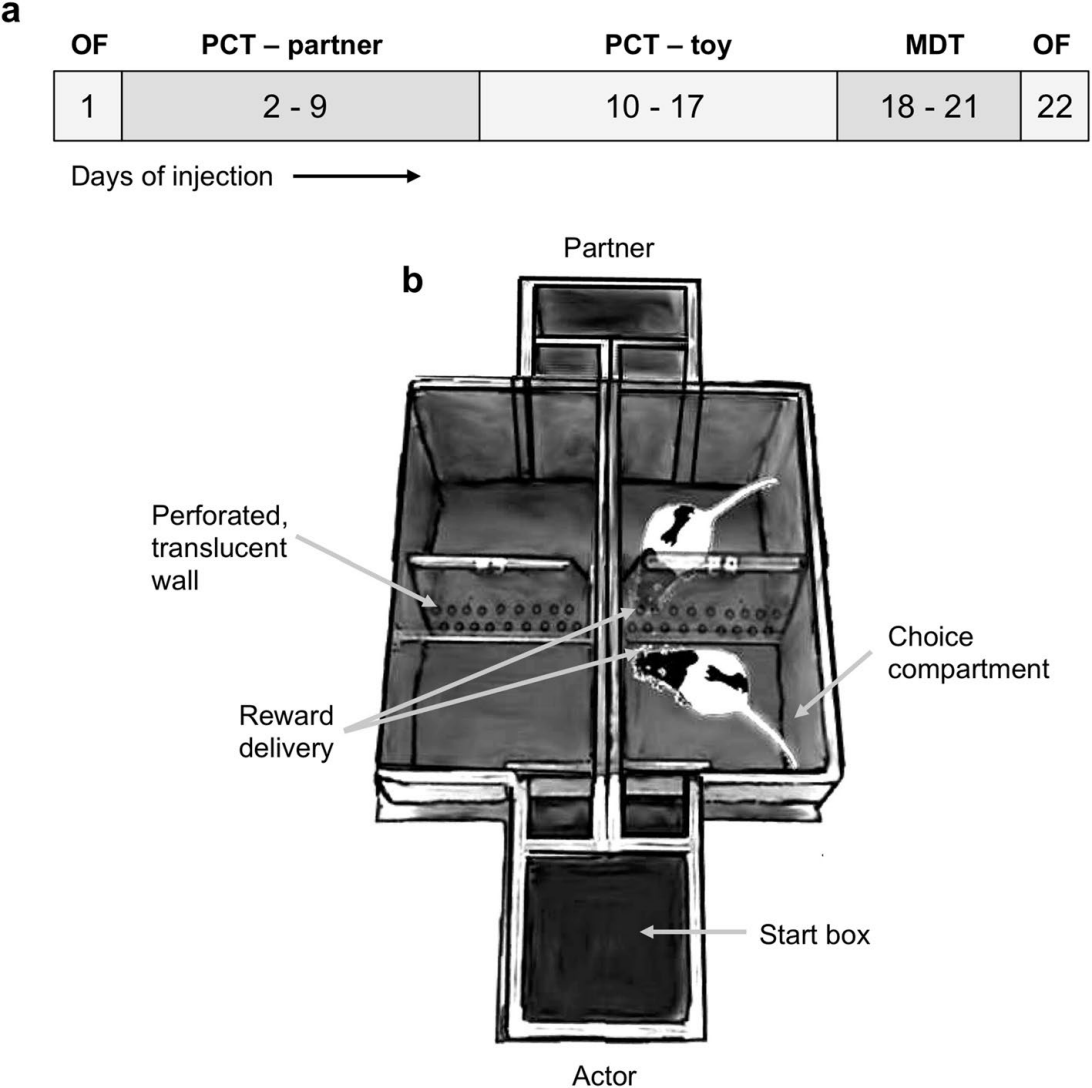
Timeline of the behavioral experiments and schematic drawing of the prosocial choice task. (**a**) The period of behavioral testing started with 1 day of open field testing, followed by 16 days of testing in the PCT (partner and toy condition lasting 8 days each), 4 days of the magnitude discrimination test and ended with another day of open field testing. Note that rats received injections of either 8-OH-DPAT or a vehicle solution shortly before each behavioral testing session. (**b**) Schematic drawing of the PCT showing actor and partner opposite to each other in the respective choice compartments; adapted from an open-access article by Hernandez-Lallement et al.^6^ distributed under the terms of the Creative Commons Attribution Licence, with friendly permission from the authors. *OF* open field, *PCT* prosocial choice task, *MDT* magnitude discrimination task.

Actor rats were randomly assigned to one of three groups, each consisting of 11 rats: group 1 received daily injections of 50 ng of the 5-HT_1A_ receptor agonist 8-OH-DPAT (Sigma-Aldrich, USA) diluted in Ringer solution, group 2 received 25 ng of the same 5-HT_1A_ receptor agonist and group 3 received a vehicle injection consisting of Ringer solution. For all injections into the BLA, a needle (C313I-SPC Internal 28GA, PlasticsOne, USA) was used that protruded 0.5 mm from the tip of the guide cannulas. During the testing period, injections were performed daily in awake rats with a 10 μl Hamilton syringe (Hamilton Company, USA) connected to a motorized injector (Harvard Apparatus, USA) at a flow rate of 0.5 μl/min. Rats received an injection volume of 0.5 μl per side and at the end of each injection the needle was left in place for 1 min to allow diffusion of the compound. Behavioral testing started 5 min after injection to ensure full pharmacological activity of the compound. In total, each rat received 22 injections of either 8-OH-DPAT or the vehicle solution; one before each of the two open field tests, one before each of the 16 sessions of the prosocial choice task (PCT) and one before each of the four magnitude discrimination task (MDT) sessions (see Fig. 1a for a complete timeline of the behavioral tests).

### Behavioral testing

#### Prosocial choice task

The apparatus and the testing paradigm of the PCT have been described in detail elsewhere^6^. Shortly, the PCT setup is a double T-maze, each consisting of a start box leading to two choice compartments on the left and the right hand side that are divided by doors from the start box. The choice compartments of the two T-mazes face each other and are separated by a perforated, translucent wall (Fig. 1b).

During behavioral testing in the PCT, two rats were present in the maze; an *actor* and a *partner*. Each actor was assigned to a partner from a different cage that was lighter than the actor and actor-partner pairs were kept constant during the entire experiment^6^. Every PCT session consisted of 6 *forced trials* and 15 *free trials*. At the beginning of a *forced trial*, the door to one of the two choice compartments was pseudorandomly opened for the actor. In a *free trial*, both doors leading to the two choice compartments were opened and the actor could freely choose to enter either compartment. One compartment was always assigned to being a *both-reward* (BR) compartment, the other compartment to being an *own-reward* (OR) compartment. Since the BR/OR-side assignment was pseudorandomized daily, the forced trials at the start of each session allowed the actor to sample the current location of the BR and OR compartments, and the number of BR-choices made in the free trials represented the behavioral readout parameter of the PCT. After the actor had entered a choice compartment, the partner had to enter the choice compartment opposite to the compartment of the actor (Fig. 1b), that is, he could never choose between the two compartments. The two rats in the opposite choice compartments could not have tactile interaction through the wall, but they could see, hear and smell each other. When both rats were located in their respective choice compartment, food rewards consisting of three sucrose pellets (45 mg; Bio Serv, USA) were delivered to one or both rats, depending on the choice of the actor. When entering the BR compartment, both actor and partner received the food reward simultaneously, when entering the OR compartment, only the actor received the food reward, but the partner did not receive any reward. The delay to reward delivery was always identical and independent from the choice of the actor. Thus, BR- and OR-choices led to identical rewards for the actor, they only differed in the payoff to the partner.

To control for secondary reinforcement effects of the pellet dropping sounds, pellet smell or other non-social factors potentially influencing the choice of the actor, a toy condition was added where a plush rat was used instead of the conspecific, which was moved into the choice compartments by the experimenter. The partner and the toy condition were identical in all other aspects, including the reward contingencies after BR- and OR-choices. Both partner and toy conditions consisted of eight testing sessions on consecutive weekdays. All actors performed both conditions; half of the actors were randomly allocated to start the experiment in the partner condition, whereas the other half started in the toy condition. Before surgery, rats were habituated to the maze and trained in a typical trial structure. After recovery from surgery, rats were re-trained followed by daily behavioral testing sessions.

Before each testing session, all actors received injections of either 8-OH-DPAT or the vehicle solution into the BLA. After the injections, the actor was placed in a cage together with its designated partner or a toy rat, to interact for 5 min prior the start of the session. The timing of the critical steps within each individual trial, including the delay to reward delivery after partner or toy had entered a choice compartment, were based on previous experiments^6,14^ and were controlled by a custom-made program using Matlab (Version 2013b; MathWorks, USA). In detail, the partner entered the opposite compartment (or the toy was placed there, respectively) 10 s after the actor. Fifteen seconds later, the sucrose pellets were dropped into both compartments after a BR-choice, or only into the compartment of the actor after an OR-choice. After reward delivery, rats had 5 s to consume the pellets, followed by a 5 s time slot for the rats to be repositioned into the start box by the experimenter and lastly, another 5 s inter-trial-interval before the start of the next trial. Thus, each trial lasted 40 s in total. As a readout parameter, BR-choices made during free trials were counted automatically for the partner and toy conditions. Furthermore, all sessions were recorded on DVD for data storage.

#### Magnitude discrimination task

To assess potential deficits in general reward learning, behavioral flexibility and spatial discrimination abilities induced by the pharmacological treatment, a magnitude discrimination task (MDT) was conducted at the end of the PCT. Only one half of the double T-maze was used, the other half was left empty. The MDT consisted of four consecutive sessions with a forced and free trials-structure identical to that of the PCT. However, neither a partner, nor a toy was present and instead of a BR and an OR side, large reward (LR) and small reward (SR) sides were pseudorandomly assigned to the choice compartments. Entering the LR side resulted in the delivery of eight sucrose pellets, whereas at the SR side only two sucrose pellets were delivered. Similar to the PCT, all actors received injections of either 8-OH-DPAT or a vehicle solution into the BLA and had to wait in a cage for 5 min before the start of the test session. Again, all sessions were recorded on DVD and LR choices during free trials were counted as behavioral readout parameter.

#### Open field test

To assess potential differences in locomotion and anxiety between the groups, an open field test was conducted on the first and last day of the pharmacological treatment period (Fig. 1a). Rats received injections of their assigned compound and after a period of 5 min were placed in the middle of a square arena (60 × 60 cm). Rats could freely explore the arena for 5 min while being observed by a camera from above. Behavioral parameters were assessed by offline analysis using tracking software (Ethovision, Noldus Information Technology, the Netherlands). The *percentage duration spent at the corners and walls* was analysed as a measure of anxiety. In addition, the parameters *distance moved* and *movement velocity* were measured to assess potential motor abnormalities induced by pharmacological treatment.

### Histology

After completion of the behavioral testing, all actor rats were transcardially perfused using 4% paraformaldehyde in 0.1 M phosphate buffer and brains were kept in the fixation solution until further processing. Using a vibratome (Leica, Germany), coronal sections at a thickness of 45 μm were cut and stained with 1% cresyl violet perchlorate in order to visualize the location of the guide cannulas. Only actors with guide cannulas targeting the BLA bilaterally were included in subsequent statistical analyses.

### Analysis

Behavioral data of the PCT were analysed using repeated measures analyses of variance (ANOVAs; SPSS 25, IBM, USA) with the three groups (50 ng OH-DPAT, 25 ng OH-DPAT or vehicle) as between-subjects factor and *condition* (partner vs. toy) as within-subjects factor (see results for more details on the analyses). Furthermore, as an additional measure of mutual-reward preferences that is more robust against individual variability^6,14^, we calculated the *social bias* (SB) score. The SB score expresses the percent difference of BR-choices in the partner relative to the toy condition, using the following equation:

$$SB = \left[ {\frac{{BR\;\left( {partner} \right) - BR\;\left( {toy} \right)}}{{BR\;\left( {toy} \right)}}} \right] \times 100.$$

SB scores greater than zero indicate more BR-choices in the partner than in the toy condition, scores equal to zero indicate equal choices in both conditions, and scores below zero more BR-choices in the toy condition. Because the reward delivered to the actor after BR- and OR-choices was always identical, SB scores higher than zero can be interpreted to reflect the added social value of a reward delivered to the conspecific^6,37,38^.

For the open field test, behavior after the first and after the last injection was compared, with *session* (acute vs. chronic) as within-subjects factor. For the MDT, a one-way ANOVA was performed to compare the percentage LR choices, averaged across the four test sessions, between the groups. Based on a conservative rule for outlier labeling^39^, outliers were identified as extreme values, which are at least three times smaller or bigger than the interquartile range, and excluded pairwise. In the main analyses, the maximum number of outliers were two cases.

## Results

### Injections of a 5-HT_1A_ receptor agonist increased mutual-reward choices in the PCT

**Figure 2:**
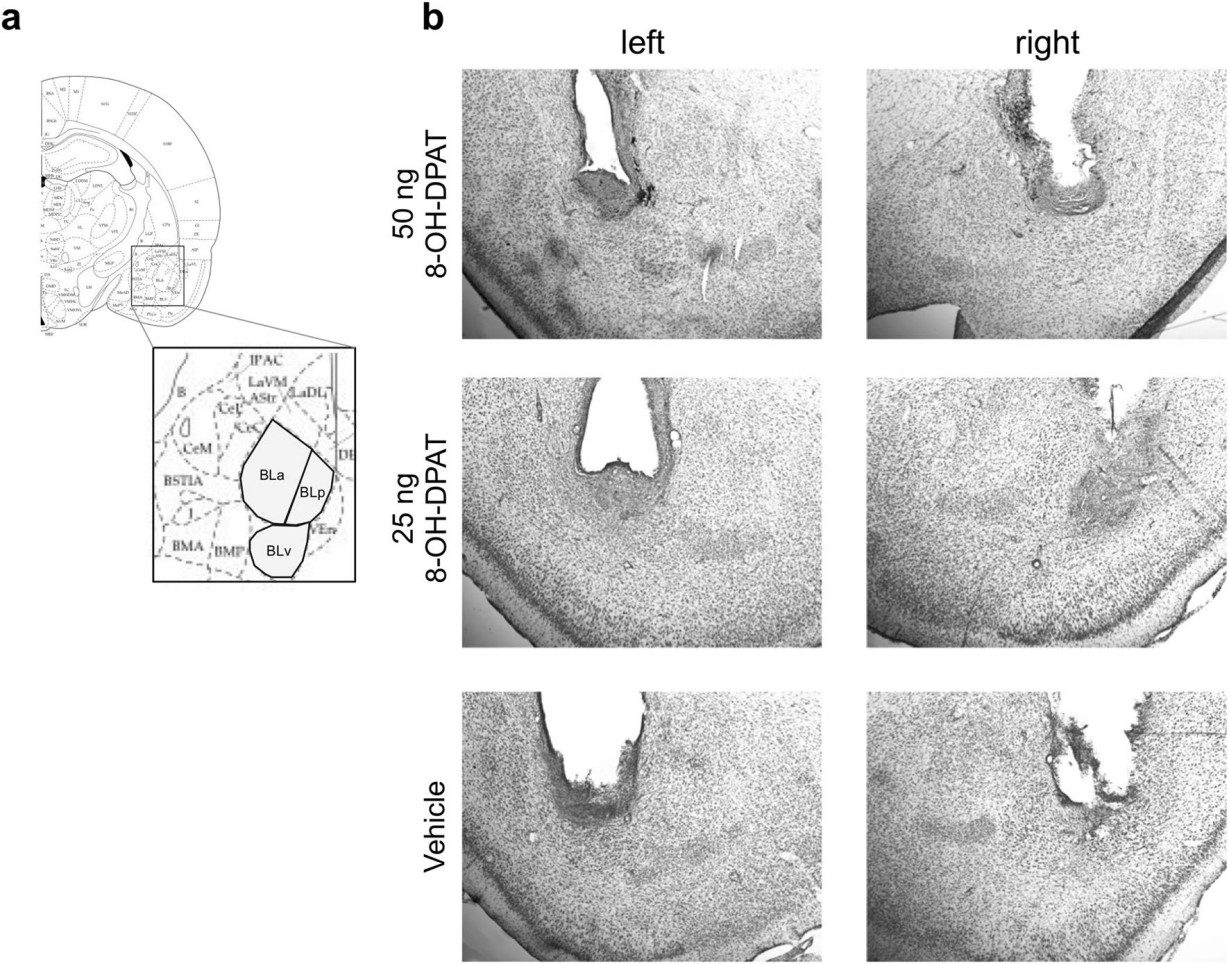
Representative examples of cannula tips in the BLA. (**a**) Magnified selection showing relevant parts of the BLA at AP 2.56 mm posterior to bregma. (**b**) Microphotographs taken at approximately AP 2.56 mm posterior to bregma showing the tips of the bilateral guide cannulas in a single microscopic slice. Guide cannulas were implanted in the BLA to allow repeated, precise injections of either 8-OH-DPAT or a vehicle solution. Please note that the respective slice of the 25 ng 8-OH-DPAT group is cut slightly asymmetrical on the AP plane. *AP* anterior–posterior, *BLA* basolateral amygdala, *BLa* anterior basolateral amygdala, *BLp* posterior basolateral amygdala, *BLv* ventral basolateral amygdala. Adapted from Paxinos and Watson^40^.

One animal had to be excluded due to bilateral misplacement of the guide cannulas (see Fig. 2 for representative examples of bilateral cannulas). As described above, the PCT consisted of eight test sessions with 15 free trials each. BR-choices were analysed in subsets of trials and sessions since we previously demonstrated that the preference for mutual rewards is learned across sessions and gradually emerges after four to five training sessions^6^. In addition, plotting the SB scores over sessions revealed a linear increase in SB scores across the first four sessions, reflecting a higher number of BR-choices in the partner relative to the toy condition, followed by stable high SB scores that did not further increase in the last four sessions in rats treated with 8-OH-DPAT (Supplementary Fig. S1).

Furthermore, we have shown that rats have to (re-)acquire a preference for the BR-side across trials in each session due to frequent BR-side reversals between sessions, and a consistent preference for the BR-side emerges after approximately ten trials into each session^7^. Therefore, we wanted to dissociate the potential effects of 8-OH-DPAT injections on learning of mutual-reward preferences from their effects on preference expression in the current study. Based on the learning curve shown in Supplementary Fig. S1 and, as previously done^6,7^, we analysed the mean percent BR-choices of the first four testing sessions (averaged across sessions 1–4; *learning phase*) and the mean percent BR-choices in the last four sessions (averaged across sessions 5–8; *expression phase*) separately. In addition, we divided the mean percent BR-choices into three blocks of trials per session and analysed them separately (block 1: trials 1–5, block 2: trials 6–10, block 3: trials 11–15; see Supplementary Fig. S2 online for the results of all possible combinations of blocks).

There was neither a significant interaction effect between condition (partner vs. toy) and group (50 ng 8-OH-DPAT, 25 ng 8-OH-DPAT or vehicle), nor significant main effects of condition or group on the percentage BR-choices detected in the learning phase (sessions 1–4) and early in a session (trial blocks 1 and 2; all p > 0.05, Supplementary Fig. S2). This suggests that 8-OH-DPAT injections did not have an effect on the acquisition speed of BR-choices and did not significantly alter reversal learning after BR-side reversals.

**Figure 3:**
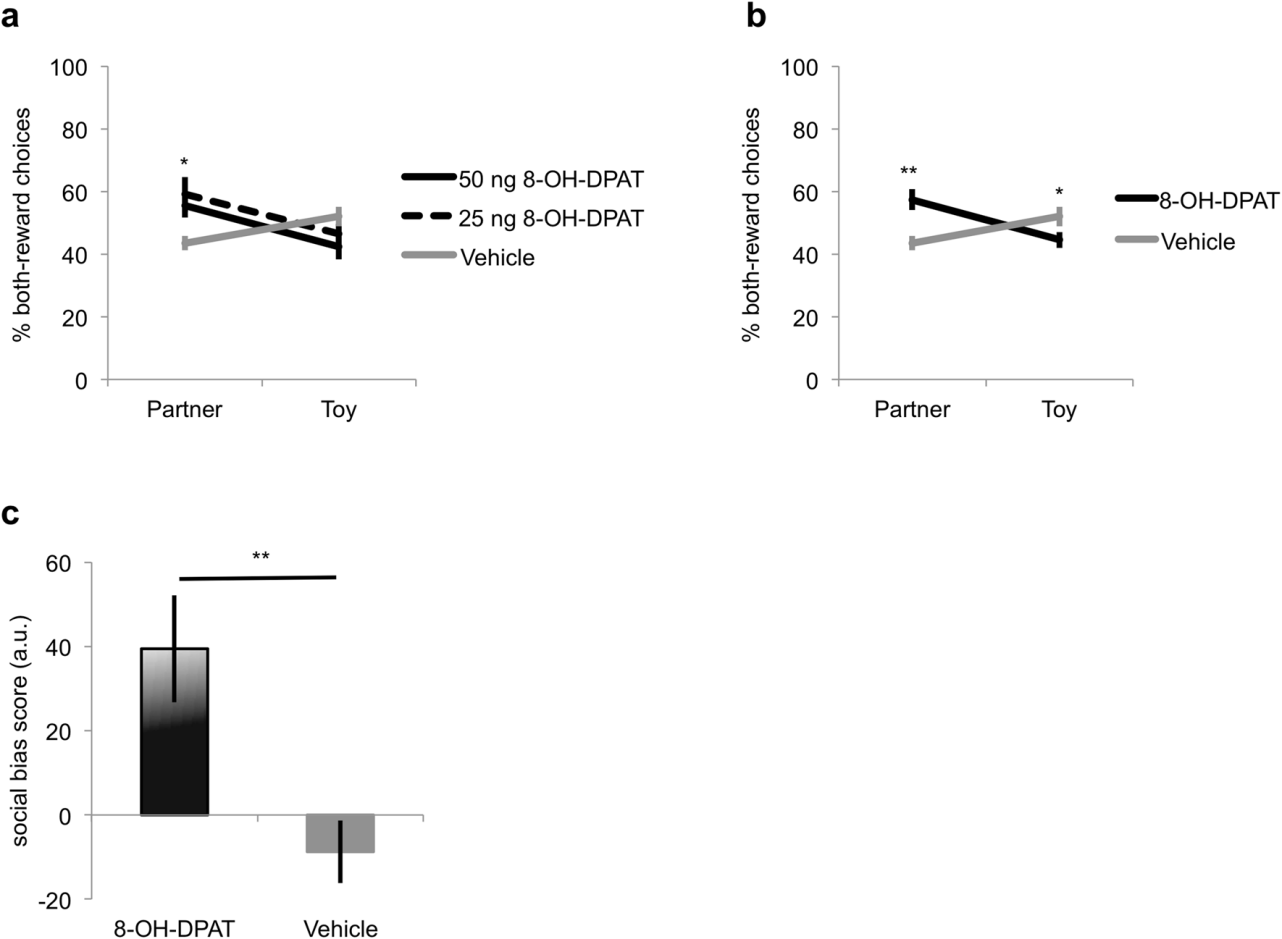
Injections of the 5-HT_1A_ receptor agonist 8-OH-DPAT increased mutual-reward choices in the prosocial choice task. This figure shows the percentage of both-reward (BR) choices and the social bias score in the expression phase (session 5–8) in block 3 (trials 11–15). (**a**) After injections of 8-OH-DPAT, rats made a higher percentage of BR-choices compared to the vehicle control group, but there were no significant differences between rats that received 50 ng or 25 ng 8-OH-DPAT. Differences between groups were only evident in the partner but not the toy condition. (**b**) Unlike vehicle injections, 8-OH-DPAT injections (50 ng and 25 ng 8-OH-DPAT groups pooled) caused an increase in BR-choices in the presence of a conspecific, but a decrease in BR-choices in the presence of a toy. (**c**) The social bias score, quantifying the normalized difference in BR-choices between the partner and the toy condition, of rats receiving 8-OH-DPAT was significantly higher compared to the social bias score of rats that received a vehicle solution. *a.u.* arbitrary units, *p < 0.05, **p < 0.01. Data are shown as mean values ± SEM.

By contrast, we found a significant interaction effect between condition and group on the percent BR-choices in the expression phase (session 5–8) once the BR-side assignment was fully learned in trial block 3 [trials 11–15; F(2,28) = 4.52, p = 0.020]. A simple effects analysis using a one-way ANOVA indicated a significant group difference in the percent BR-choices in the partner [F(2,30) = 3.74, p = 0.036], but not in the toy condition [F(2,31) = 2.00, p = 0.154; Fig. 3a]. Post-hoc contrasts did not reveal a significant difference in the percent BR-choices between the 50 ng and 25 ng 8-OH-DPAT groups [t(17.32) = − 0.53, p = 0.600], but the percent BR-choices in both 8-OH-DPAT groups together was significantly different from the percent BR-choices in the vehicle group [t(26.19) = 3.41, p = 0.002]. These results suggest that 8-OH-DPAT injections into the BLA increased BR-choices relative to vehicle, but without a significant difference in BR-choices between the 50 ng and 25 ng group. For further analysis, we pooled the data of two 8-OH-DPAT groups and we weighted the data of the vehicle group with a factor 2 and the data of the pooled 8-OH-DPAT group with a factor 1 to simulate equal sample sizes.

To further explore the differences in the 8-OH-DPAT injection effects on BR-choices between the partner and the toy condition, we ran a second repeated measures ANOVA, again on the data of the expression phase and trial block 3 (session 5–8, trials 11–15). A significant interaction effect between condition and group (8-OH-DPAT vs. vehicle) on the percent BR-choices was found [F(1,39) = 17.14, p = 0.000]. Again, the analysis of the simple effects showed a significant difference between the two groups in the partner condition [F(1,40) = 13.61, p = 0.001], but also a difference between the two groups in the toy condition [F(1,42) = 5.31, p = 0.026; Fig. 3b]. This result corroborates the previous conclusion that, relative to rats receiving vehicle injections, rats receiving 8-OH-DPAT injections made significantly more BR-choices in the partner condition. In addition, rats injected with 8-OH-DPAT made significantly less BR-choices in the toy condition compared to rats that received vehicle injections.

We also tested if the percent BR-choices were significantly different from chance (50%) in the pooled 8-OH-DPAT and vehicle group. A one sample t-test revealed that rats receiving 8-OH-DPAT injections made BR-choices significantly above chance in the partner condition [t(20) = 2.19, p = 0.040] and significantly below chance in the toy condition [t(20) = − 2.17, p = 0.043]. The vehicle group, in turn, made BR-choices significantly below chance in the partner condition [t(19) = − 4.21, p = 0.000] and their percent BR-choices did not differ significantly from chance level in the toy condition [t(21) = 0.94, p = 0.358].

To quantify the effects of 8-OH-DPAT on the vicarious social value of a reward delivered to the partner, we analysed the SB scores using the same subset of data (sessions 5–8, trials 11–15). We found a significant difference in the SB scores between the pooled 8-OH-DPAT group and the vehicle group [F(1,42) = 12.75, p = 0.001; Fig. 3c], suggesting that 8-OH-DPAT injections boosted vicarious reward value processing. Taken together, our results imply that 8-OH-DPAT injections increased mutual-reward choices during later sessions and trials, independent of dosage, but there was no evidence to assume that 8-OH-DPAT injections affected the acquisition of BR preferences in early sessions or the speed of reversal learning.

In all rats the vertically implanted cannulas left visible tracts in the brain regions above the BLA that corresponded to the actual cannula diameter. At the end points of the guide cannulas, slight cellular abnormalities were observed that morphologically could be classified as astrogliosis and presumably occurred as a result of repeated penetration by the injection needle. It is important to note that we did not observe differences in the amount of damage to the brain parenchyma between rats in all three groups. In addition, there was no significant decline in performance in all rats over the testing sessions, which speaks against a predominant influence of tissue damage on mutual-reward choices [F(7,175) = 1.1; p = 0.37]. We therefore concluded that putative lesion effects on behavior were negligible. In addition, the fact that we found differences in mutual-reward choices between experimental and control rats despite an identical injection procedure suggests that the injection effects on behavior cannot be attributed to putative tissue damage, but are likely due to the active compound 8-OH-DPAT*.*

### Injections of a 5-HT_1A_ receptor agonist did not alter reward learning, anxiety or locomotion

All actors were tested in the MDT where they had to decide between a compartment with a large reward (LR) or a compartment with a small reward (SR). A one-way ANOVA with group (50 ng 8-OH-DPAT, 25 ng 8-OH-DPAT or vehicle) as between-subjects factor on the percentage LR-choices did not show a significant difference between the three groups [F(2,30) = 0.25; p = 0.784; Supplementary Fig. S3a]. All groups had a strong preference for the LR side, which was significantly higher than chance set at 50% (all groups p < 0.001).

The open field test was performed at the first and last day of the injection period to measure any changes in locomotion or anxiety resulting from either acute or chronic administration of 8-OH-DPAT. A repeated measures ANOVA with session (acute vs. chronic) as within-subjects factor and group (50 ng 8-OH-DPAT, 25 ng 8-OH-DPAT or vehicle) as between-subjects factor showed that animals significantly decreased the percentage duration spent close to the corners and walls of the arena [F(1,27) = 5.90; p = 0.022; Supplementary Fig. S3b] without any significant differences between the groups [F(1,27) = 0.05; p = 0.949], indicating a decrease in anxiety during exploration, regardless of the treatment. Also, the total distance moved [F(1,28) = 35.36; p = 0.000; Supplementary Fig. S3c] and the movement velocity [F(1,28) = 35.39; p = 0.000; Supplementary Fig. S3d] increased over time, probably due to body growth, without any significant differences between the groups (both p = 0.490). Overall, these additional parameters demonstrate that injections of 8-OH-DPAT did not cause any unspecific effects that might confound behavior in the PCT.

## Discussion

In the present study, we investigated the role of 5-HT_1A_ receptors in the BLA on mutual-reward choices in rats. We administered repeated intracerebral injections of the 5-HT_1A_ receptor agonist 8-OH-DPAT into the BLA and performed behavioral testing in the PCT. In this task, actor rats made non-costly choices that either yielded a reward to themselves and a conspecific (both-reward choices; BR), or only to themselves and not to the conspecific (own-reward choices; OR). We found that, relative to injections with a vehicle solution, injections of 8-OH-DPAT increased the number of BR-choices when the rats were paired with a conspecific, and decreased the number of BR-choices when paired with a non-social toy rat. 8-OH-DPAT injections did not alter locomotion or anxiety. Our finding converges with and expands previous research from our group that suggests that mutual-reward preferences in untreated rats are BLA-dependent^6,14^ and mediated by serotonergic neurotransmission in the BLA.

Serotonin plays a role in affective and social processes and is dysregulated in depression, anxiety, autism spectrum disorder and many more^41–43^. It was shown in a recent study that serotonin concentrations in human saliva were negatively correlated with feelings of empathy and social sharing of happiness^44^, yet in another study participants became harm-avoidant after an acute dose of the SSRI citalopram^19^. In rodents, systemically administered cannabidiol decreased aggressive behavior towards a conspecific after a period of social isolation, whereas systemic MDMA increased social interaction, both effects being mediated by 5-HT_1A_ receptors^45,46^. Finally, social isolation has been shown to broadly interfere with normal serotonergic signaling in rats^25,26^.

The BLA receives a substantial number of serotonergic projections from the dorsal raphe nuclei and expresses different types of 5-HT receptors. Interestingly, it has been shown that injections of the 5-HT_1A_ agonist 8-OH-DPAT into the BLA decreased social investigation in rats^47^. Based on our findings, it is tempting to speculate that agonistic action on 5-HT_1A_ receptors in the BLA caused an increased sensitivity towards social signals and a more careful selection of appropriate behaviors, in line with human studies where systemic serotonin modulation increased awareness of harming others and the sensitivity to unfair behavior^18–20^. Here, rats treated with 8-OH-DPAT made significantly more choices that resulted in a food reward to a conspecific partner; at the same time, they opted significantly less often for the delivery of the same food reward to a toy rat. This divergent decision-making might reflect a deliberate selection of social behavior in a social situation versus non-social behavior in a non-social situation^48^, which might explain why rats treated with 8-OH-DPAT made more BR-choices in the presence of a partner and more OR-choices in the presence of a toy.

Our results support the idea that the BLA integrates social information and contributes to the selection of appropriate social actions. We have recently argued that this selection of social actions might be the result of social reinforcement learning^7,14,37,38,49^. According to this framework, BR-choices of the actor might prompt the emission of appetitive social signals by the partner rat, which in turn reinforce the behaviors of the actor associated with these signals, eventually increasing the probability of BR-choices. By contrast, OR-choices by the actor might trigger aversive social signals by the partner and actors might consequently avoid behaviors associated with those signals, thus decreasing the probability of OR-choices. In rats, potential candidate signals that might launch social reinforcement processes are ultrasonic vocalizations^49–52^. Our main finding that 5-HT_1A_ receptor agonists in the BLA increase mutual-reward choices is consistent with this framework. Serotonergic neurotransmission in the BLA might increase the sensitivity to the rewarding aspects of social signals by amplifying their incentive value and, by consequence, their motivational effects on social behavior. Future research should be conducted to further characterize the interplay between serotonin in the amygdala and the emission of ultrasonic vocalizations, as examples of social reinforcers, and their moderating role in social behavior.

The finding of increased mutual-reward choices after 8-OH-DPAT injections is inconsistent with previous observations where comparable doses led to increased anxiety and less interest in social interaction^30,47^. In our study, we did not find any difference in anxiety-like behavior between groups following injections of either 8-OH-DPAT or vehicle in the open field test, therefore the effect of 8-OH-DPAT injections on mutual-reward choices was most likely not due to alterations in anxiety or locomotion. While being contrary to the results of Gonzalez et al.^47^, our findings are in line with a recent study reporting an increase in social interaction after 8-OH-DPAT injections^53^. However, the experiments show marked methodological differences as in the first study, low doses of the respective agonist (50 ng and 200 ng) were injected solely into the BLA, whereas in the second study, much higher doses (60 μg/kg and 125 μg/kg) were administered systemically.

From our previous studies, we know that healthy rats need several testing sessions to acquire a stable level of BR-choices^6^ and in the present study, we found an effect of 8-OH-DPAT only in late testing sessions (session 5–8, expression phase), but not in early testing sessions (session 1–4, learning phase), suggesting that 8-OH-DPAT has different effects on learning and expression of mutual-reward choices. Repeated stimulation of serotonergic neurotransmission has been associated with neurogenesis and neuroplasticity^54–56^, which might be a factor mediating the increased sensitivity towards social choices after 8-OH-DPAT injections, both in the partner and in the toy condition. Furthermore, repeated injections of 8-OH-DPAT were associated with improved spatial learning in rats suffering from a mechanical brain injury^57^. Interestingly, spatial learning of the rats injected with 8-OH-DPAT was superior to the sham group only after the sixth testing session, but not at the beginning of the behavioral test^57^, which fits our observation that behavioral group differences in the PCT were only present during later testing sessions.

Previous research by our group has shown that there are considerable individual differences in mutual-reward preferences between rats^6^ that potentially dilute the group means of BR choices. This suggests that our results are presumably at the lower limit of the true choice distribution, implying that we possibly underestimated the true effect size of the 8-OH-DPAT injections on mutual-reward preferences. One important aspect where the present study differs from our previous work is that the current control group did not make more mutual-reward choices in the partner compared to the toy condition. In our previous experiments, untreated and sham operated rats had a preference for the BR side in the presence of a conspecific, but no specific side preference in the presence of a toy rat^6,14^. However, the behavior exhibited by rats in the current vehicle group should not be compared one-on-one to previous control groups, since rats from the vehicle group underwent the same kind of implantation surgery as the groups receiving 8-OH-DPAT injections. The mere presence of the cannulas might lead to inflammatory reactions of the surrounding tissue, which might even be exaggerated by the mechanical and psychological stress of daily injections. Recently it was shown that the insertion of an injection needle through a guide cannula into the BLA was sufficient to trigger the occurrence of an acute spreading depolarization that in turn caused freezing behavior and lasted for 1.5 min after the initial insult^58^. In addition, BLA lesions have been specifically associated with decreased mutual-reward choices in the PCT^14^ and do interfere with general reward learning in multiple ways (reviewed in Wassum and Izquierdo^59^). Although group differences in the amount of overt tissue damage were absent (Fig. 2b), we cannot rule out the possibility that small residual, undetected amounts of tissue damage in the BLA might have subtly altered the processing of and reaction to social stimuli, which in the vehicle group were not buffered by pharmacological treatment. In the two other groups the pharmacological treatment could additionally have spread to and thereby affect neighboring regions, such as the basomedial amygdala, that also plays a role in social behavior^60^. However, we consider this possibility negligible, due to correct cannula placement and the small injection volume. Finally, injections of isotonic solutions might alter the amount of local signaling molecules, leading to slight changes in neurotransmission or even behavioral effects^61^ and therefore are not equivalent to no-treatment. In conclusion, all of these factors might have added noise to the behavior of our rats in the PCT, thus masking mutual-reward preferences that are already weak in untreated animals to begin with.

Another aspect worth mentioning is the fact that the 5-HT_1A_ receptor acts as both an inhibitory autoreceptor at serotonergic postsynapses terminating in the BLA and as an inhibitory heteroreceptor at GABAergic and glutamatergic neurons in the BLA. Therefore, an agonist to the 5-HT_1A_ receptor could decrease serotonergic input from the dorsal raphe nucleus, decrease the output of GABAergic inhibitory interneurons, decrease the output of glutamatergic excitatory pyramidal neurons or a combination of all three. Despite the latter heterogeneity of net effects caused by 5-HT_1A_ receptor agonism, we still found a clear effect of 5-HT_1A_ receptor activation in the partner and in the toy condition where agonist injections increased, respectively decreased, social responses in rats.

In conclusion, we have shown that injections of an agonist of the 5-HT_1A_ receptor into the BLA caused an increase in mutual-reward choices, without non-specific effects on anxiety or locomotion. Our results provide a first indication of the relevance of the serotonergic system in the amygdala, specifically the widely distributed 5-HT_1A_ receptor, for social behavior in rats. Future experiments are necessary using a 5-HT_1A_ receptor antagonist on its own and in combination with 8-OH-DPAT to validate the current effect of 5-HT_1A_ receptor agonism on the behavior of rats in the PCT. Furthermore, experiments are needed in which 5-HT_1A_ receptors are targeted on distinct cell types to specify the exact impact of 5-HT in the amygdala on social behavior.

## Supplementary information

Supplementary Figures.

## Data Availability

The datasets generated and analysed during the current study are available from the corresponding author on reasonable request.

## References

[1] Barnett SA (1958). An analysis of social behaviour in wild rats. J. Zool..

[2] Schneeberger K, Dietz M, Taborsky M (2012). Reciprocal cooperation between unrelated rats depends on cost to donor and benefit to recipient. BMC Evol. Biol..

[3] Takano Y, Ukezono M, Nakashima SF, Takahashi N, Hironaka N (2017). Learning of efficient behaviour in spatial exploration through observation of behaviour of conspecific in laboratory rats. R. Soc. Open Sci..

[4] Zentall TR, Levine JM (1972). Observational learning and social facilitation in the rat. Science.

[5] Bartal IB-A, Decety J, Mason P (2011). Empathy and pro-social behavior in rats. Science.

[6] Hernandez-Lallement J, van Wingerden M, Marx C, Srejic M, Kalenscher T (2015). Rats prefer mutual rewards in a prosocial choice task. Front. Neurosci..

[7] Hernandez-Lallement J, van Wingerden M, Schäble S, Kalenscher T (2017). A social reinforcement learning hypothesis of mutual reward preferences in rats. Curr. Topics Behav. Neurosci..

[8] Marquez C, Rennie SM, Costa DF, Moita MA (2015). Prosocial choice in rats depends on food-seeking behavior displayed by recipients. Curr. Biol..

[9] Chang SW, Fagan NA, Toda K, Utevsky AV, Pearson JM, Platt ML (2015). Neural mechanisms of social decision-making in the primate amygdala. Proc. Natl. Acad. Sci. USA.

[10] Diergaarde L, Gerrits MA, Stuy A, Spruijt BM, van Ree JM (2004). Neonatal amygdala lesions and juvenile isolation in the rat: Differential effects on locomotor and social behavior later in life. Behav. Neurosci..

[11] Felix-Ortiz AC, Tye KM (2014). Amygdala inputs to the ventral hippocampus bidirectionally modulate social behavior. J. Neurosci..

[12] Liu X (2018). Neuroimaging studies reveal the subtle difference among social network size measurements and shed light on new directions. Front. Neurosci..

[13] Daenen EW, Wolterink G, Gerrits MA, Van Ree JM (2002). The effects of neonatal lesions in the amygdala or ventral hippocampus on social behaviour later in life. Behav. Brain Res..

[14] Hernandez-Lallement J, van Wingerden M, Schäble S, Kalenscher T (2016). Basolateral amygdala lesions abolish mutual reward preferences in rats. Neurobiol. Learn. Mem..

[15] Salomon RM, Cowan RL (2013). Oscillatory serotonin function in depression. Synapse.

[16] Wolf D (2018). Central serotonin modulates neural responses to virtual violent actions in emotion regulation networks. Brain Struct. Funct..

[17] Balazsfi D (2018). Differential roles of the two raphe nuclei in amiable social behavior and aggression—An optogenetic study. Front. Behav. Neurosci..

[18] Crockett MJ (2013). Serotonin modulates striatal responses to fairness and retaliation in humans. J. Neurosci..

[19] Crockett MJ, Clark L, Hauser MD, Robbins TW (2010). Serotonin selectively influences moral judgment and behavior through effects on harm aversion. Proc. Natl. Acad. Sci. USA.

[20] Crockett MJ, Clark L, Tabibnia G, Lieberman MD, Robbins TW (2008). Serotonin modulates behavioral reactions to unfairness. Science.

[21] Homberg JR, Schiepers OJ, Schoffelmeer AN, Cuppen E, Vanderschuren LJ (2007). Acute and constitutive increases in central serotonin levels reduce social play behaviour in peri-adolescent rats. Psychopharmacology.

[22] Knutson B, Panksepp J (1997). Effects of serotonin depletion on the play of juvenile rats. Ann. NY Acad. Sci..

[23] Raleigh MJ, McGuire MT, Brammer GL, Pollack DB, Yuwiler A (1991). Serotonergic mechanisms promote dominance acquisition in adult male vervet monkeys. Brain Res..

[24] Arborelius L, Eklund MB (2007). Both long and brief maternal separation produces persistent changes in tissue levels of brain monoamines in middle-aged female rats. Neuroscience.

[25] Dalley JW, Theobald DE, Pereira EA, Li PM, Robbins TW (2002). Specific abnormalities in serotonin release in the prefrontal cortex of isolation-reared rats measured during behavioural performance of a task assessing visuospatial attention and impulsivity. Psychopharmacology.

[26] Han X, Wang W, Shao F, Li N (2011). Isolation rearing alters social behaviors and monoamine neurotransmission in the medial prefrontal cortex and nucleus accumbens of adult rats. Brain Res..

[27] Asan E, Steinke M, Lesch KP (2013). Serotonergic innervation of the amygdala: Targets, receptors, and implications for stress and anxiety. Histochem. Cell Biol..

[28] Bonn M, Schmitt A, Lesch KP, Van Bockstaele EJ, Asan E (2013). Serotonergic innervation and serotonin receptor expression of NPY-producing neurons in the rat lateral and basolateral amygdaloid nuclei. Brain Struct. Funct..

[29] Palchaudhuri M, Flugge G (2005). 5-HT1A receptor expression in pyramidal neurons of cortical and limbic brain regions. Cell Tissue Res..

[30] Strauss CV, Vicente MA, Zangrossi H (2013). Activation of 5-HT1A receptors in the rat basolateral amygdala induces both anxiolytic and antipanic-like effects. Behav. Brain. Res..

[31] Sardari M, Rezayof A, Zarrindast MR (2015). 5-HT1A receptor blockade targeting the basolateral amygdala improved stress-induced impairment of memory consolidation and retrieval in rats. Neuroscience.

[32] Bizot J, Le Bihan C, Puech AJ, Hamon M, Thiebot M (1999). Serotonin and tolerance to delay of reward in rats. Psychopharmacology.

[33] Centenaro LA (2008). Social instigation and aggressive behavior in mice: Role of 5-HT1A and 5-HT1B receptors in the prefrontal cortex. Psychopharmacology.

[34] Evenden JL (1999). The pharmacology of impulsive behaviour in rats VII: The effects of serotonergic agonists and antagonists on responding under a discrimination task using unreliable visual stimuli. Psychopharmacology.

[35] Picazo O, Lopez-Rubalcava C, Fernandez-Guasti A (1995). Anxiolytic effect of the 5-HT1A compounds 8-hydroxy-2-(di-n-propylamino) tetralin and ipsapirone in the social interaction paradigm: Evidence of a presynaptic action. Brain Res. Bull..

[36] OECD. Guidance Document on the Recognition, Assessment, and Use of Clinical Signs as Humane Endpoints for Experimental Animals Used in Safety Evaluation vol ENV/JM/MONO(2000)7. Paris (2000).

[37] Hernandez-Lallement J, van Wingerden M, Kalenscher T (2018). Towards an animal model of callousness. Neurosci. Biobehav. Rev..

[38] van Gurp S, Hoog J, Kalenscher T, van Wingerden M (2019). Social value unblocks Pavlovian reinforcement learning in male rats. bioRxiv.

[39] Hoaglin DC, Iglewicz B, Tukey JW (1986). Performance of some resistant rules for outlier labeling. J. Am. Stat. Assoc..

[40] Paxinos G, Watson C (1997). The Rat Brain in Stereotaxic Coordinates.

[41] Kraus C, Castren E, Kasper S, Lanzenberger R (2017). Serotonin and neuroplasticity—Links between molecular, functional and structural pathophysiology in depression. Neurosci. Biobehav. Rev..

[42] Muller CL, Anacker AMJ, Veenstra-VanderWeele J (2016). The serotonin system in autism spectrum disorder: From biomarker to animal models. Neuroscience.

[43] Olivier B (2015). Serotonin: A never-ending story. Eur. J. Pharmacol..

[44] Matsunaga, M. et al. Association between salivary serotonin and the social sharing of happiness. *PLoS One* **12**(7), e0180391 (2017). 10.1371/journal.pone.0180391 PMC5500317 28683075

[45] Hartmann A (2019). Cannabidiol attenuates aggressive behavior induced by social isolation in mice: Involvement of 5-HT1A and CB1 receptors. Prog. Neuropsychopharmacol. Biol. Psychiatry.

[46] Hunt GE, McGregor IS, Cornish JL, Callaghan PD (2011). MDMA-induced c-Fos expression in oxytocin-containing neurons is blocked by pretreatment with the 5-HT-1A receptor antagonist WAY 100635. Brain Res. Bull..

[47] Gonzalez LE, Andrews N, File SE (1996). 5-HT1A and benzodiazepine receptors in the basolateral amygdala modulate anxiety in the social interaction test, but not in the elevated plus-maze. Brain Res..

[48] Kiser D, Steemers B, Branchi I, Homberg JR (2012). The reciprocal interaction between serotonin and social behaviour. Neurosci. Biobehav. Rev..

[49] Kalenscher, T. Rat ultrasonic vocalizations as social reinforcers—Implications for a multilevel model of the cognitive representation of action and rats' social world. *Proceedings in Cognitive Structures* (2020).

[50] Burgdorf J, Knutson B, Panksepp J, Ikemoto S (2001). Nucleus accumbens amphetamine microinjections unconditionally elicit 50-kHz ultrasonic vocalizations in rats. Behav. Neurosci..

[51] Kashtelyan V, Lichtenberg NT, Chen ML, Cheer JF, Roesch MR (2014). Observation of reward delivery to a conspecific modulates dopamine release in ventral striatum. Curr. Biol..

[52] Willuhn I (2014). Phasic dopamine release in the nucleus accumbens in response to pro-social 50 kHz ultrasonic vocalizations in rats. J. Neurosci..

[53] Depoortere R, Bardin L, Varney MA, Newman-Tancredi A (2019). Serotonin 5-HT1A receptor biased agonists display differential anxiolytic activity in a rat social interaction model. ACS Chem. Neurosci..

[54] Banasr M, Hery M, Printemps R, Daszuta A (2004). Serotonin-induced increases in adult cell proliferation and neurogenesis are mediated through different and common 5-HT receptor subtypes in the dentate gyrus and the subventricular zone. Neuropsychopharmacology.

[55] Guirado R (2012). Chronic fluoxetine treatment in middle-aged rats induces changes in the expression of plasticity-related molecules and in neurogenesis. BMC Neurosci..

[56] Kodama M, Fujioka T, Duman RS (2004). Chronic olanzapine or fluoxetine administration increases cell proliferation in hippocampus and prefrontal cortex of adult rat. Biol. Psychiat..

[57] Mala H (2013). Only repeated administration of the serotonergic agonist 8-OH-DPAT improves place learning of rats subjected to fimbria-fornix transection. Pharmacol. Biochem. Behav..

[58] Vinogradova LV, Rysakova MP, Pavlova IV (2020). Small damage of brain parenchyma reliably triggers spreading depolarization. Neurol. Res..

[59] Wassum KM, Izquierdo A (2015). The basolateral amygdala in reward learning and addiction. Neurosci. Biobehav. Rev..

[60] Mesquita LT (2016). New insights on amygdala: Basomedial amygdala regulates the physiological response to social novelty. Neuroscience.

[61] Ikeda H, Kotani A, Koshikawa N, Cools AR (2007). A vehicle injection into the right core of the nucleus accumbens both reverses the region-specificity and alters the type of contralateral turning elicited by unilateral stimulation of dopamine D2/D3 and D1 receptors in the left core of the nucleus accumbens. Eur. J. Pharmacol..

