# Supplementary material for: 5-HT1A receptor agonism in the basolateral amygdala increases mutual-reward choices in rats
Source: Sci Rep. 2020 Oct 6;10:16622. doi: 10.1038/s41598-020-73829-z (PMC7538979; doi:10.1038/s41598-020-73829-z)
Supplement: Supplementary file 1 — Supplementary Figures. [file 41598_2020_73829_MOESM1_ESM.pdf]

$5\text{-}HT_{1A}$ receptor agonism in the basolateral amygdala increases mutual-reward choices in rats
Scientific Reports

Lisa-Maria Schönfeld, Sandra Schäble, Maurice-Philipp Zech & Tobias Kalenscher*
* Comparative Psychology, Institute of Experimental Psychology, Heinrich Heine University Düsseldorf, 40225 Düsseldorf, Germany; Tobias.Kalenscher@hhu.de

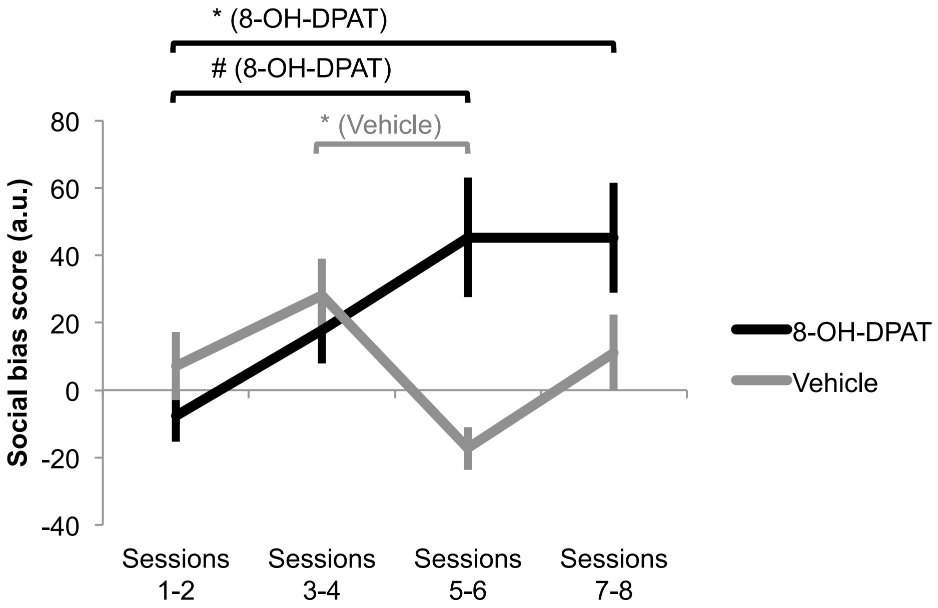

Supplementary Figure S1
Social bias scores of the group receiving 8-OH-DPAT injections and the vehicle group plotted over pairs of sessions. In the 8-OH-DPAT group, social bias scores linearly increase during the first sessions and reach a plateau at the last four sessions, indicating an increase in the number of both-reward choices in the partner condition compared to the toy condition. On the contrary, social bias cores in the vehicle group fluctuate around zero, indicating equal amounts of BR-choices in the partner and in the toy condition.
* $p < .05$; # $p > .05$ and $p < .07$

5-HT$_{1A}$ receptor agonism in the basolateral amygdala increases mutual-reward choices in rats
Scientific Reports

Lisa-Maria Schönfeld, Sandra Schäble, Maurice-Philipp Zech & Tobias Kalenscher*
* Comparative Psychology, Institute of Experimental Psychology, Heinrich Heine University Düsseldorf, 40225 Düsseldorf, Germany; Tobias.Kalenscher@hhu.de

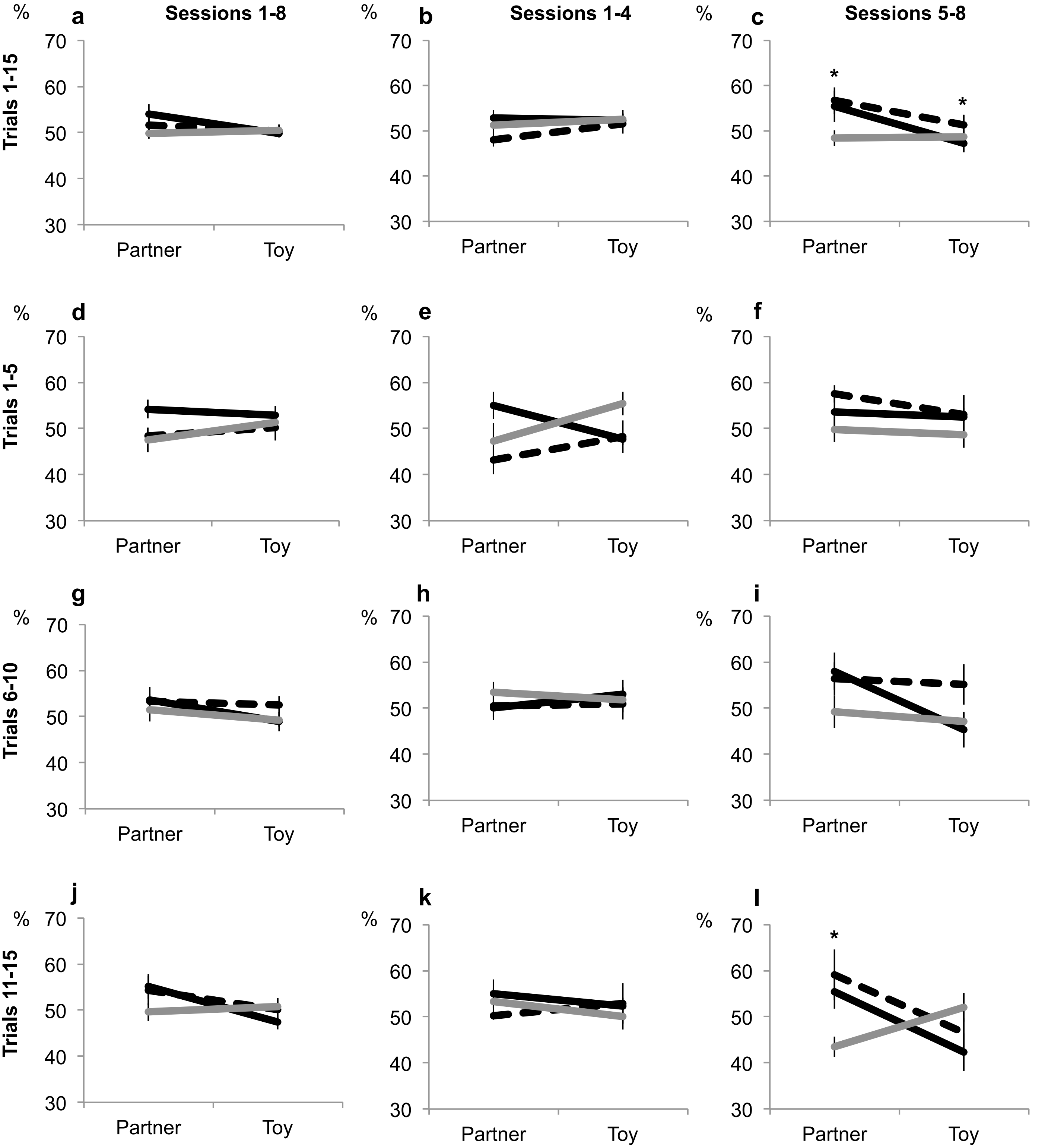

Supplementary Figure S2
All combinations of sessions and trial blocks. * p< .05

Lisa-Maria Schönfeld, Sandra Schäble, Maurice-Philipp Zech & Tobias Kalenscher*
* Comparative Psychology, Institute of Experimental Psychology, Heinrich Heine University Düsseldorf, 40225 Düsseldorf, Germany; Tobias.Kalenscher@hhu.de

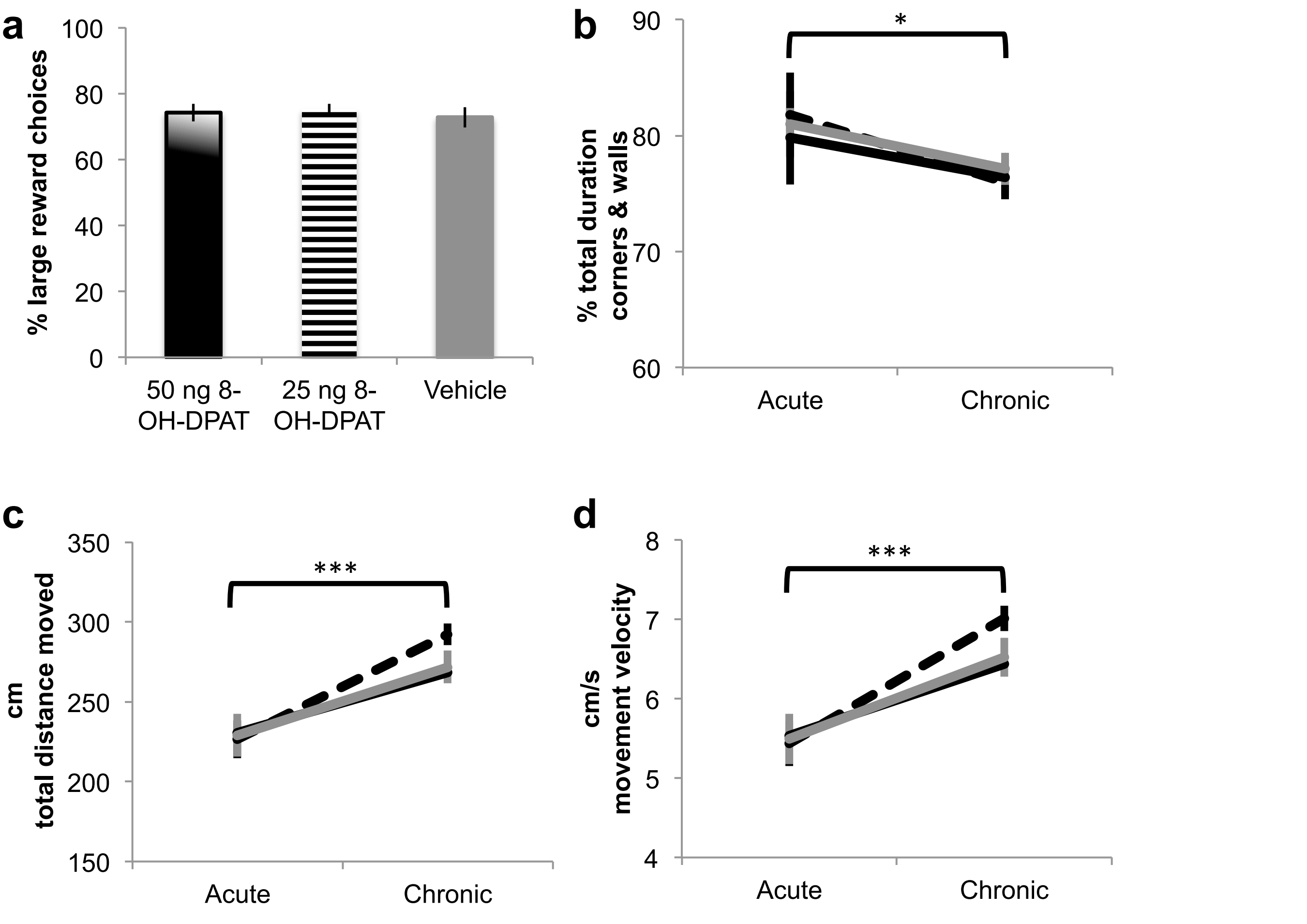

Supplementary Figure S3
Treatment with the 5-HT$_{1A}$ receptor agonist 8-OH-DPAT did not affect reward learning, behavioral flexibility, anxiety and locomotion.
* $p < .05$; *** $p < .001$
